# On the Antioxidant Properties of L-Kynurenine: An Efficient ROS Scavenger and Enhancer of Rat Brain Antioxidant Defense

**DOI:** 10.3390/antiox11010031

**Published:** 2021-12-24

**Authors:** Daniela Ramírez Ortega, Perla Eugenia Ugalde Muñiz, Tonali Blanco Ayala, Gustavo Ignacio Vázquez Cervantes, Rafael Lugo Huitrón, Benjamín Pineda, Dinora Fabiola González Esquivel, Gonzalo Pérez de la Cruz, José Pedraza Chaverrí, Laura Sánchez Chapul, Saúl Gómez-Manzo, Verónica Pérez de la Cruz

**Affiliations:** 1 Neurochemistry and Behavior Laboratory, National Institute of Neurology and Neurosurgery “Manuel Velasco Suárez”, Mexico City 14269, Mexico; drmz_ortega@hotmail.com (D.R.O.); tonaliblaya@gmail.com (T.B.A.); guigvace@gmail.com (G.I.V.C.); dinora.gonzalez@innn.edu.mx (D.F.G.E.); 2 Laboratorio de Neuroendocrinología, Facultad de Medicina, Universidad Nacional Autónoma de México (UNAM), Mexico City 04510, Mexico; perlaugalde@ciencias.unam.mx; 3 Laboratorio de Neurobiología Conductual, Facultad de Medicina, Universidad Nacional Autónoma de México (UNAM), Mexico City 04510, Mexico; lugorafael@ciencias.unam.mx; 4 Neuroimmunology Department, National Institute of Neurology and Neurosurgery “Manuel Velasco Suárez”, Mexico City 14269, Mexico; benjamin.pineda@innn.edu.mx; 5 Department of Mathematics, Faculty of Sciences, Universidad Nacional Autónoma de México (UNAM), Mexico City 04510, Mexico; gonzalo.perez@ciencias.unam.mx; 6 Department of Biology, Faculty of Chemistry, Universidad Nacional Autónoma de México (UNAM), Mexico City 04510, Mexico; pedraza@unam.mx; 7 Neuromuscular Diseases Laboratory, National Institute of Rehabilitation “Luis Guillermo Ibarra Ibarra”, Mexico City 14389, Mexico; lausanchez@inr.gob.mx; 8 Laboratorio de Bioquímica Genética, Instituto Nacional de Pediatría, Secretaría de Salud, Mexico City 04530, Mexico; saulmanzo@ciencias.unam.mx

**Keywords:** kynurenine, redox environment, antioxidant, scavenger, kynurenine pathway, reactive oxygen species, glutathione, glutathione peroxidase, brain, neuroprotection

## Abstract

L-kynurenine (L-KYN) is an endogenous metabolite, that has been used as a neuroprotective strategy in experimental models. The protective effects of L-KYN have been attributed mainly to kynurenic acid (KYNA). However, considering that L-KYN is prone to oxidation, this redox property may play a substantial role in its protective effects. The aim of this work was to characterize the potential impact of the redox properties of L-KYN, in both synthetic and biological systems. First, we determined whether L-KYN scavenges reactive oxygen species (ROS) and prevents DNA and protein oxidative degradation in synthetic systems. The effect of L-KYN and KYNA (0.1–100 µM) on redox markers (ROS production, lipoperoxidation and cellular function) was compared in rat brain homogenates when exposed to FeSO_4_ (10 µM). Then, the effect of L-KYN administration (75 mg/kg/day for 5 days) on the GSH content and the enzymatic activity of glutathione reductase (GR) and glutathione peroxidase (GPx) was determined in rat brain tissue. Finally, brain homogenates from rats pretreated with L-KYN were exposed to pro-oxidants and oxidative markers were evaluated. The results show that L-KYN is an efficient scavenger of ^●^OH and ONOO^−^, but not O_2_^●–^ or H_2_O_2_ and that it prevents DNA and protein oxidative degradation in synthetic systems. L-KYN diminishes the oxidative effect induced by FeSO_4_ on brain homogenates at lower concentrations (1 µM) when compared to KYNA (100 µM). Furthermore, the sub-chronic administration of L-KYN increased the GSH content and the activity of both GR and GPx, and also prevented the oxidative damage induced by the ex vivo exposure to pro-oxidants. Altogether, these findings strongly suggest that L-KYN can be considered as a potential endogenous antioxidant.

## 1. Introduction

**Figure 1:**
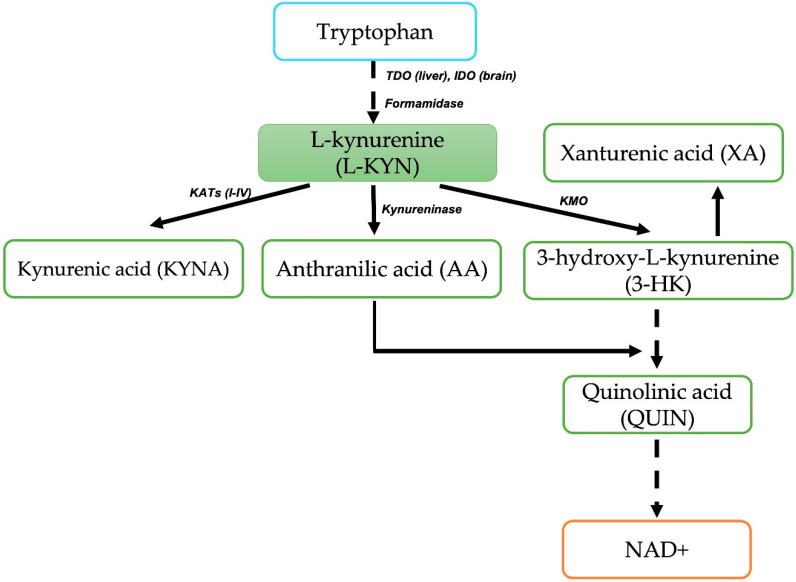
Kynurenine Pathway. The major catabolic route of tryptophan metabolism showing the main metabolites and enzymes conducing to the essential enzyme cofactor NAD+. TDO = tryptophan 2,3-dioxygenase; IDO = indoleamine 2,3-dioxygenase; KAT’s = kynurenine aminotransferases; KMO = kynurenine-3-monooxygenase; NAD+ = nicotinamide adenine dinucleotide.

L-kynurenine (L-KYN) is an intermediate metabolite of the kynurenine pathway (KP). In mammals, the KP is the primary catabolic route for L-tryptophan (TRP), representing approximately 95% of the total TRP catabolism, leading to the formation of various metabolites known as kynurenines and ultimately producing the essential cofactor for cellular redox reactions, nicotinamide adenine dinucleotide (NAD+) [1,2] (Figure 1). KP metabolites have physiological and pathological relevance due to their neuroactive and redox properties [3,4,5,6,7,8,9,10,11,12]. The KP initiates when TRP is converted to N-formyl-L-kynurenine by the hepatic tryptophan 2,3-dioxygenase (TDO) and the extrahepatic indoleamine 2,3-dioxygenase (IDO), which catalyze the oxidative cleavage of TRP; next, L-KYN is produced by the action of kynurenine formamidase [13,14] (Figure 1). It has been estimated that nearly 60% of the total L-KYN found in the brain derives from the periphery since L-KYN can cross the blood–brain barrier through the Na^+^-independent transporter of neutral amino acids [15]. Once formed, L-KYN is a substrate for different KP enzymes: kynureninase to produce anthranilic acid (AA); kynurenine-3-monooxygenase (KMO) to produce 3-hydroxykynurenine (3-HK), and kynurenine aminotransferases (KATs) to generate kynurenic acid (KYNA). KP continues from both 3-HK and AA being enzymatically converted to 3-hydroxyanthranilic acid, which is further transformed by 3-hydroxyanthranilic oxidase (3-HAOO) into 2-amino-3-carboxymuconic-6-semialdehyde, an unstable intermediate that spontaneously rearranges to form quinolinic acid (QUIN). Finally, QUIN is further metabolized by quinolinate phosphoribosyltransferase for the synthesis of NAD+.

The neuroprotective effects of L-KYN have been previously shown in different experimental models. L-KYN has been frequently used as an in vivo pharmacological tool to rapidly increase brain KYNA levels due to its ability to cross the blood–brain barrier (BBB). Less is known about its direct protective effects within the brain apart from KYNA. However, evidence has been provided over the years that gives some clues about an independent and possibly, an additional, effect of KYNA production.

The neuroprotective effect L-KYN was shown in a neonatal rat model (7-days old, Sprague-Dawley) of hypoxia–ischemia. Intraperitoneal administration of L-KYN (50–300 mg/kg) 1 h before hypoxia–ischemia dose-dependently reduced the infarct rate and infarct volume in the brain tissue. However, it is interesting to notice that KYNA levels measured within the brain cortex after the administration of increasing doses of L-KYN were not significantly different, while its neuroprotective effect was shown to be dose-dependent. Additional effects of L-KYN were also demonstrated; L-KYN pretreatment reduced in a dose-dependent manner the c-fos protein expression in the cerebral hemisphere ipsilateral to the carotid ligation. This protein is rapidly and transiently expressed in response to several stimuli including brain injury and ischemia. *N*-methyl-d-aspartate receptor (NMDAr) antagonists can block c-fos expression; thus, L-KYN administration was used for increasing KYNA levels and inducing NMDAr inhibition. L-KYN (300 mg/kg) almost completely blocked the expression of c-fos protein but without increasing KYNA levels to the optimal inhibitory concentration (EC_50_: 10–30 μM) reported for NMDAr [16]. More recently, it was shown that the intraperitoneal administration of L-KYN (300 mg/kg) decreased the number of injured neurons in the cortex of pre- and post-treated ischemic rats. They provided the first histological proof of the neuroprotective effect of L-KYN in the cortex after global ischemia in adult rats. In this study, the authors suggested that systemic administration of L-KYN offers advantages over KYNA derivatives in the treatment of ischemic brain damage [17]. In an additional study, L-KYN prevented the ischemia-induced deterioration of spatial memory (spontaneous alternation) and attenuated hypermotility in gerbils. L-KYN (30 mg/kg, i.p.) administration (only 15 min before focal cerebral ischemia) in mice induced a small decrease in the surface of the brain infarction; this low dose of L-KYN does not normally induce a substantial increase in cerebral KYNA. Furthermore, it is argued that only very high doses of KYNA (800 mg/kg) are known to exert a neuroprotective effect in these experimental models while lower doses were ineffective, while a neuroprotective effect of L-KYN was demonstrated from lower doses [18,19].

On the other hand, following the rationale of attributing L-KYN effects to the increase of KYNA and its inhibitory action on NMDAr. The effect of sub-chronic administration of L-KYN (75 mg/kg/day, i.p.; 7 days) alone or with probenecid (PROB) (50 mg/kg/day, i.p.; 7 days)—an inhibitor of organic acids transport—was tested in a rat model of soluble amyloid beta (Aβ) toxicity [20]. The authors did not find differences between L-KYN and L-KYN+ PROB groups, in both groups there were significant protection against the deleterious effects provoked by Aβ, therefore suggesting that they exerted protection at a similar magnitude. In this study, no motor alterations were found after the sub-chronic administration of L-KYN or L-KYN+PROB, either after 30 min or ~1.5 h (the latter being the maximum time reported for the maximum increase in KYNA after systemic administration of L-KYN). Likewise, findings from another research group showed that KYNA i.v. injection induced alterations in locomotor and vertical activities and increased stereotyped activity accompanied by ataxia [21]. Interestingly, these deleterious effects were not induced by L-KYN administration; this discrepancy between KYNA and L-KYN effects suggests additional mechanisms of L-KYN prior to being metabolized through the KP.

Additionally, the ability to reduce oxidative damage following a treatment combination of L-KYN+PROB has been shown in QUIN and 6-hydroxydopamine (6-OHDA) toxicity models [22,23,24]. Specifically, it was pointed out that 6-OHDA auto-oxidation can directly generate ROS, mostly H_2_O_2_ and ^•^OH, as well as O_2_^●−^ leakage from Complex I of the mitochondrial respiratory chain; thus, it was suggested that the protective effects of L-KYN+PROB treatment could be related to the antioxidant properties of KYNA as a hydroxyl scavenger, reducing ROS formation and preventing 6-OHDA auto-oxidation [24]. Therefore, the importance of redox modulation in the neuroprotective effects following L-KYN administration is emphasized. The antioxidant profile for L-KYN has been previously shown in non-tissue systems; an electrochemical study showed that the presence of the amino group in the L-KYN structure facilitates auto-oxidation at high potentials [25]. Furthermore, it was shown that L-KYN can scavenge hydroxyl radicals and reduce luminol chemiluminescence in vitro when hydrogen peroxide or chloramine T was added to the medium in a dose-dependent manner [26].

Considering the above background, this work focuses on elucidating the redox properties of L-KYN in synthetic (combinatory chemistry) and biological systems (in vitro and ex vivo). Here we first determined in synthetic (non-tissue) systems the redox scavenging ability of L-KYN and then explored whether this could be partially independent of KYNA scavenging properties; for this purpose, a KYNA antibody was used to capture the KYNA produced by the direct interaction between L-KYN and ROS to minimize its antioxidant effect. In biological systems, L-KYN and KYNA were used at the same concentrations when exposed to pro-oxidants to compare the extent of the protective effect of each metabolite against oxidative damage in rat brain tissues. Then, we determined whether this protective effect of L-KYN could be reproduced in vivo using a sub-chronic administration of L-KYN to subsequently evaluate the redox environment in the rat brain tissues under basal and oxidative conditions.

## 2. Materials and Methods

### 2.1. Reagents

2′,7′-dichlorofluorescein (DCF), DCF-diacetate (DCF-DA), L-KYN, 3-nitropropionic acid (3-NP), FeSO_4_, xylenol orange, ammonium iron (II) sulfate hexahydrate, 2,2′-azinobis-(3-ethylbenzothiazoline-6-sulfonic acid) (ABTS), 2,2-diphenyl-1-picrylhydrazyl (DPPH), bovine serum albumin (BSA), nitroblue tetrazolium (NBT), phenazine methosulfate (PMS), ethylenediaminetetraacetic acid (EDTA), nicotinamide adenine dinucleotide (NADH), diethylenetriaminepentaacetic acid (DTPA), sodium hypochlorite (NaOCl), H_2_O_2_ and *N*,*N*-dimethyl-4-nitrosoaniline (DMNA) were all obtained from Sigma Chemical Company (St. Louis, MO, USA). All other reagents were reactive grade and obtained from known commercial suppliers. Solutions were prepared using deionized water obtained from a Milli-RQ (Millipore, Burlington, MA, USA) purifier system.

### 2.2. Animals

Tissues were obtained from the whole brains of male Wistar rats (280–320 g) from the vivarium of the National Institute of Neurology (Mexico City, Mexico). Before the experiments, the animals were housed in acrylic cages and were provided with a standard commercial rat diet (Laboratory rodent diet 5001, PMI Feeds Inc., Richmond, IN, USA) and water ad libitum. The housing room was maintained under constant conditions of temperature (25 ± 3 °C), humidity (50 ± 10%), and lighting (12 h light/dark cycles). Tissues were collected by decapitation, immediately dissected on ice, and preserved for a limited time at −70 °C. All animal procedures were carried out according to the National Institutes of Health Guide for the Care and Use of Laboratory Animals and the local guidelines on the ethical use of animals from the Health Ministry of Mexico.

### 2.3. Chemical Combinatory Assays

#### 2.3.1. Superoxide Radicals Scavenging Assessment

O_2_^●−^ scavenging was assessed according to a previously reported method [27] based on the reduction of the NBT. The non-enzymatic PMS/NADH system generates superoxide radicals that reduce NBT into a purple-colored formazan. The reaction mixture was composed of HEPES buffer (20 mM, pH 7.4), 196 µM NADH, 39.2 µM NBT, and 3.92 µM PMS, where we tested increasing concentrations of L-KYN (1, 2.5, 5, 10, 25 and 50 µM). The final mixture volume was 1.3 mL. After incubation for 5 min at room temperature, the absorbance for each sample was determined at 560 nm. All tests were independently performed six times. The data are shown as the percent of O_2_^●−^ scavenging capacity.

#### 2.3.2. Hydroxyl Radical Scavenging Assay

The ability of L-KYN (1, 2.5, 5, 10, 25 and 50 µM) to scavenge ^●^OH was estimated using the Fe^3+^-EDTA-H_2_O_2_-deoxyribose system [28,29]. This system contained different concentrations of L-KYN (or an equivalent volume of distilled water for the control), 0.2 mM ascorbic acid, 0.2 mM FeCl_3_, 0.208 mM EDTA, 1 mM H_2_O_2_, 0.56 mM deoxyribose, and 20 mM phosphate buffer (pH 7.4). Hydroxyl radicals were generated after incubating the mixture at 37 °C for 60 min. The iron salt (FeCl_3_) was mixed with EDTA before its addition to the reaction mixture. The amount of deoxyribose degradation by ^●^OH was measured directly in the aqueous phase by the thiobarbituric acid (TBA) test. Briefly, 100 µL of TBA solution was added to the samples and final solutions were boiled in a water bath (94 °C) for 10 min. The optical density was determined at a wavelength of 532 nm in a Genesys 8 spectrophotometer. The results are shown as percent of •OH scavenging capacity.

#### 2.3.3. ONOO^−^ Scavenging Activity Assessment

ONOO^−^ was synthesized as previously described [30]. Five milliliters of an acidic solution (0.6 M HCl) of H_2_O_2_ (0.7 M) were mixed with 5 mL of 0.6 M KNO_2_ on an ice bath for 1 s and the reaction was quenched with 5 mL of ice-cold 1.2 M NaOH. Residual H_2_O_2_ was removed using granular MnO_2_ pre-washed with 1.2 M NaOH and then the reaction mixture was left overnight at −20 °C. The resulting yellow liquid layer on the top of the frozen mixture was collected. The final concentrations of ONOO^−^ were determined before each experiment at 302 nm using a molar extinction coefficient of 1670 M^−1^cm^−1^. ONOO^−^ scavenging activity was measured by monitoring the oxidation of DCF-DA to DCF by the modified method of Crow and Beckman [31]. The reaction mixture (in a final volume of 1.5 mL in 0.1 M phosphate buffer pH 7.4) contained 14 µM DTPA and 36.2 µM DCF-DA; samples were incubated with different L-KYN concentrations (1, 2.5, 5, 10, 25 and 50 µM) and exposed to 35 µM ONOO^−^. The optical density was determined at 500 nm in a Genesys 8 spectrophotometer (Thermo Scientific, Waltham MA, USA). A standard containing only the reaction mixture was considered as 0% scavenging capacity or 100% of DCF-DA oxidation induced by the ONOO^−^ added to the assay. To calculate the ONOO^−^ scavenging ability, the optical density values for those samples with L-KYN were expressed as a percentage of DCF-DA oxidation and converted to a percentage of scavenging ability, using the standard tube as 100% of DCF-DA oxidation reference. Before determining the ability of L-KYN to scavenge ONOO^−^ apart from KYNA formation we used a polyclonal KYNA antibody (Abcam, ab37105). For this purpose, we added 0.1 µL of KYNA antibody (0.5 mg/µL) and 2.2 µM of L-KYN or 600 µM KYNA to the reaction mixture, and the optical density was determined after 3 min of reaction.

#### 2.3.4. Hydrogen Peroxide Assay

The ability of L-KYN to scavenge H_2_O_2_ was measured by the method described by Long et al. (1999) [32]. Briefly, 9 volumes of 4.4 mM butylated hydroxytoluene in HPLC-grade methanol were mixed with 1 volume of 1 mM xylenol orange and 2.56 mM ferrous ammonium sulfate in 0.25 M H_2_SO_4_ to give the “working” FOX reagent. A solution of 75 μM H_2_O_2_ was mixed with different concentrations of L-KYN (1, 2.5, 5, 10, 25 and 50 µM) and 0.01 mL of HPLC-grade methanol, followed by the immediate addition of 0.9 mL of FOX reagent. The solutions were then mixed for 5 s and incubated at room temperature for 30 min. The tubes were centrifuged at 15,000× *g* for 10 min, and absorbance was read at 560 nm against methanol blank [32].

#### 2.3.5. ^•^OH-Mediated Protein Degradation

Experiments performed for the detection of ^•^OH-mediated oxidation of bovine-serum albumin (BSA) were carried out by using a metal-catalyzed reaction based on the method described by Kocha et al. (1997) with modifications [33,34]. A solution of ascorbic acid (1.6 mM)/EDTA (0.8 mM)/(NH_4_)_2_ Fe(SO_4_)_2_ (0.8 mM) was prepared in 50 mM phosphate buffer (pH 7.4) plus BSA and L-KYN. Briefly, 1% of BSA was mixed with 250 μL of the ascorbic acid/EDTA/(NH_4_)_2_ Fe(SO_4_)_2_ solution, in the presence or absence of L-KYN (0.1, 1, 5, 10, 20, 50 μM). The generation of ^•^OH was initiated through the addition of 15 µL of 2% H_2_O_2_. In control tubes (without the ^•^OH generator system), H_2_O_2_ was replaced by water. After 1 h of incubation at room temperature, 250 μL of 20% trichloroacetic acid was added, and the mixture was then centrifuged at 2200× *g* for 30 min at 4 °C. The supernatants were discarded, and the pellets were re-suspended in 500 μL of 0.1 M NaOH. The samples were prepared for SDS-polyacrylamide gel electrophoresis to evaluate the oxidative damage induced to proteins induced by ^•^OH. BSA (50 μg from re-suspended pellet) was mixed (1:1) with loading buffer (10% glycerol, 2% SDS, 25 mM Tris-HCl (pH 6.8), 5% mercaptoethanol, 0.1% bromophenol blue) and heated at 100 °C for 1 min. The protein sample was loaded in a 12% polyacrylamide gel, and electrophoresis was performed at 150 V for 1 h. After the electrophoresis, gels were stained with 0.2% Coomassie brilliant blue R for 1 h. Images were visualized and captured in a BioRad Gel Documentation System (Gel Doc 1000, BioRad, Hercules, CA, USA). Protein levels were evaluated by densitometry assessment using the Quantity One Program 4.2 (BioRad, Hercules, CA, USA).

#### 2.3.6. ^•^OH-Mediated DNA Degradation

DNA samples were obtained from three adult FVB mice tails and incubated overnight at 55 °C in lysis buffer (Tris 100 mM (pH 8.5), 5 mM EDTA, 0.2% SDS, 200 mM NaCl and 100 µg/mL proteinase K). The mixtures were deproteinized with phenol:chloroform:isoamyl alcohol (25:24:1). The DNA in the supernatant was precipitated by adding cold isopropanol and washed with 70% ethanol solution. It was then dissolved in endonucleases-free water for the assay. Experiments for the detection of ^●^OH-mediated DNA oxidation were carried out using a metal-catalyzed reaction, which was also used for protein degradation. Briefly, 20 µg of purified DNA was mixed with 25 µL of the ascorbic acid/EDTA/(NH_4_)_2_Fe(SO_4_)_2_ solution, in the presence or absence of L-KYN. The generation of ^●^OH was initiated through the addition of 15 μL of 2% H_2_O_2_. In control tubes (without the ^●^OH generator system), H_2_O_2_ was replaced by water. The final volume for all probes was 250 µL. After 15 min of remaining at room temperature, 50 μL of loading buffer was added to stop the reaction. To evaluate the oxidative damage induced by ^●^OH to DNA, 10 µL of the samples were loaded in an agarose (2%) gel and electrophoresis was performed for 20 min at 90 V. Then, agarose gels were stained with ethidium bromide (0.1 mg/mL). Images were visualized and captured in a BioRad Gel Documentation System (Gel Doc 1000 BioRad). To estimate DNA degradation, each DNA band was evaluated by densitometry using the Quantity One Program 4.2.

### 2.4. In Vitro Assays

#### 2.4.1. In Vitro Incubation

Briefly, brain tissue from five animals per group were homogenized (1:10 *w*/*v*;) in Krebs buffer (118.5 mM NaCl; 4.75 mM KCl; 1.77 mM CaCl_2_; 1.18 mM MgSO_4_; 5 mM glucose; 12.9 mM NaH_2_PO_4_; and 3 mM Na_2_HPO_4_; pH 7.4). Then, 375 µL of brain homogenate was incubated alone or with FeSO_4_ (10 µM) in the presence of L-KYN (0–100 µM) or KYNA (0–100 µM) in a final volume of 500 µL for 2 h at 37 °C in a water bath. After incubation, the samples were used for simultaneous assays for ROS, lipid peroxidation (LP) and MTT reduction.

#### 2.4.2. Determination of ROS Levels in Homogenates

ROS were evaluated through DCF-DA oxidation [35]. Briefly, 125 µL of forebrain homogenates previously incubated with FeSO_4_ and kynurenines were mixed with DCF-DA solution (final concentration: 75 µM), then incubated under dark conditions at 37 °C for 30 min. After incubation, the samples were centrifuged at 9000× *g* for 10 min. ROS were detected in supernatants using fluorescence spectrophotometry (Synergy™ HTX multi-mode microplate reader, Biotek Instruments, Winooski, VT, USA) at an excitation wavelength of 480 nm and an emission wavelength of 521 nm. Results are expressed as a percentage of ROS production considering the control as 100%.

#### 2.4.3. Determination of Lipid Peroxidation (LP) in Homogenates

According to a previous report, LP was assessed in forebrain homogenates by the determination of TBA-reactive substances (TBA-RS) [35]. After incubation with FeSO_4_ and kynurenines, 125 µL of the brain homogenate were mixed with 250 μL of the TBA reagent (containing 0.375 g of TBA + 15 g of trichloroacetic acid + 2.54 mL of HCl) and incubated in a boiling water bath (94 °C) for 20 min. Samples were then kept on ice and centrifuged at 12,000× *g* for 5 min. Malondialdehyde (MDA) was determined as a colorimetric product using Synergy™ HTX multi-mode microplate reader (Biotek Instruments, Winooski, VT, USA) at a wavelength of 532 nm. Results were expressed as a percentage of TBA-RS production considering the control as 100%.

#### 2.4.4. Determination of Cellular Function in Homogenates

The cellular function was assessed in forebrain homogenates by determining MTT reduction to formazan crystals [36,37]. Briefly, the brain homogenate (125 µL) was mixed with 4 μL of the MTT (5 mg/mL) and then incubated for 15 min at 37 °C. Samples were then centrifuged at 17,000× *g* for 3 min and the pellets were suspended in 250 μL with acid-isopropanol. Optical density was determined using an Eon microplate reader (Biotek Instruments, Winooski, VT, USA) at a wavelength of 570 nm. Results are expressed as the percentage of MTT reduction considering the control as 100%.

### 2.5. Ex Vivo Assays

#### 2.5.1. L-KYN Administration and Ex Vivo Incubation with Pro-Oxidants

Five rats per group were intraperitoneally (i.p.) administrated with L-KYN (75 mg/kg) or saline solution daily at 8 a.m. for 5 days. Then, the rats were euthanized by decapitation after the last i.p. injection, and their brains were rapidly harvested. Brain tissue was homogenized (1:10, *w*/*v*) in Krebs buffer (pH 7.4). Then, 375 µL of each brain homogenate was incubated alone or with FeSO_4_ (10 µM), 3-NP (2 mM) or ONOO^−^ (50 µM) in a final volume of 500 µL for 2 h at 37 °C in a water bath. After incubation, the sample was used for the simultaneous determination of ROS, LP and MTT reduction as previously described. In separate brain homogenates, GSH and GSSG levels, GSH reductase and GSH peroxidase enzymatic activity, and kynurenines levels were determined for each group.

#### 2.5.2. GSH and GSSG Content

For the determination of GSH and GSSG levels, brain tissue was weighed immediately after harvesting and then homogenized (1:10, *w*/*v*) in buffer A (containing: 154 mM KCl, 5 mM DTPA, and 0.1 M potassium phosphate buffer, pH 6.8). After being homogenized the brain tissue was mixed with the same amount of cold buffer B (containing: 40 mM HCl, 10 mM DTPA, 20 mM ascorbic acid and 10% trichloroacetic acid) as buffer A, and centrifuged (14,000× *g*, 20 min). Supernatants were collected and filtered through a syringe filter with a 0.22 µm pore size hydrophilic PES membrane. For GSH determination, 5 µL of the supernatant was mixed with o-phthaldialdehyde (OPA) to obtain the isoindole. For GSSG determination, 30 µL of the supernatant was mixed with 4 µL of N-ethylmaleimide (7.5 mM final concentration) to neutralize GSH. Subsequently, GSSG was reduced to GSH using 6 µL of sodium hydrosulfite (100 mM final concentration). Finally, derivatization with OPA was performed to obtain the isoindole. The isoindole was measured by fluorescence at 370 nm (excitation) and 420 nm (emission) using a Synergy™ HTX multi-mode microplate reader (Biotek Instruments, Winooski, VT, USA) [35]. Results are expressed as nanomoles of GSH or GSSG/g of tissue.

#### 2.5.3. Glutathione Reductase (GR) Activity

Glutathione reductase (GR) activity was measured by monitoring NADPH consumption [38]. Brains from control and L-KYN groups were homogenized 1:10 (*w*/*v*) in a buffer containing 3.09 µM KH_2_PO_4_, 1.90 µM Na_2_HPO_4_, 0.05% Triton 100×, pH 7, then samples were centrifuged at 10,000× *g* for 30 min. The collected supernatants were used to determine GR activity. Briefly, 16.5 µL of the supernatant samples were added to 300 µL of the reaction cocktail (1.90 µM Na_2_HPO_4_, 0.62 mM EDTA, 0.11 mM NADPH, 1.2 mM GSSG in KH_2_PO_4_ buffer, pH 7.4), and D.O. at 340 nm was acquired every min for 3 min. GR specific activity was calculated as the slope of the change of D.O. of NADPH consumption and expressed as U/mg protein.

#### 2.5.4. Glutathione Peroxidase (GPx) Activity

GPx activity was measured using a modified spectrophotometric method [39,40]. The reaction mixture was made in KH_2_PO_4_ buffer (100 mM KH_2_PO_4_; 1 mM EDTA) pH 7.4 by adding glutathione reductase (1 U/mL), GSH (1 mM) and NADPH (0.2 mM). The reaction mixture (200 µL) and 25 µL of a sample (dilution 1:10) were added to each plate well. The reaction was initiated by the addition of 100 µL of H_2_O_2_ (0.25 mM). The absorbance values at 340 nm were recorded for 5 min every 30 s. GPx activity was calculated from the slopes, using the molar absorptivity for NADPH (6.22 × 103 M^−1^cm^−1^). GPx activity was expressed as U/mg protein.

#### 2.5.5. Kynurenine Levels

The tissues were thawed and homogenized in deionized water (1:10, *w*/*v*) to determine total L-KYN, KYNA, and 3-HK levels. Thirty microliters of perchloric acid (6%) was added to the samples and then centrifuged (14,600× *g*, 10 min). The resulting supernatant was used for HPLC analysis [35].

For L-KYN and KYNA, 50 µL was applied to a 3 µm C_18_ reverse-phase column (ZORBAX Eclipse XDB 5 µm, 4.6 mm × 150 mm; Agilent, Santa Clara, CA, USA), and metabolites were isocratically eluted using a mobile phase containing 250 mM zinc acetate, 50 mM sodium acetate and 3% acetonitrile (pH 6.2) at a flow rate of 1 mL/min. In the eluate, KYNA was detected fluorometrically (excitation wavelength: 344 nm; emission wavelength: 398 nm) with a retention time of ~7 min. L-KYN was detected at excitation wavelength: 368 nm; emission wavelength: 480 nm using an S200a fluorescence detector (Perkin-Elmer, Waltham, MA, USA). The retention time for L-KYN was ~6 min.

For 3-HK, 100 µL of the resulting supernatant was applied to a 3-µm Adsorbosphere C18 reverse-phase column (4.6 mm × 100 mm; Fisher Scientific, Hampton, NH, USA), using a mobile phase consisting of 1.5% acetonitrile, 0.9% triethylamine, 0.59% phosphoric acid, 0.27 mM EDTA, and 8.9 mM sodium heptane sulfonic acid, and a flow rate of 0.6 mL/min. The reaction product, 3-HK, was detected electrochemically using a LC-4C detector (BAS, West Lafayette, IN, USA) oxidation potential: +0.5 V) in the eluate. The retention time for 3-HK was ~11 min.

#### 2.5.6. Protein Assay

Protein was determined according to the Lowry method [41] using bovine serum albumin as protein standard.

#### 2.5.7. Statistical Analysis

All data are expressed as the mean ± SEM. Two-way ANOVA was performed using the Kruskal–Wallis test with Dunn’s test for multiple pairwise comparisons. When comparing only two groups a Mann–Whitney test was performed. *p*-values lower than *p* < 0.05 were considered significant.

## 3. Results

### 3.1. Scavenging Activity of L-KYN in Combinatorial Chemistry Assays

**Figure 2:**
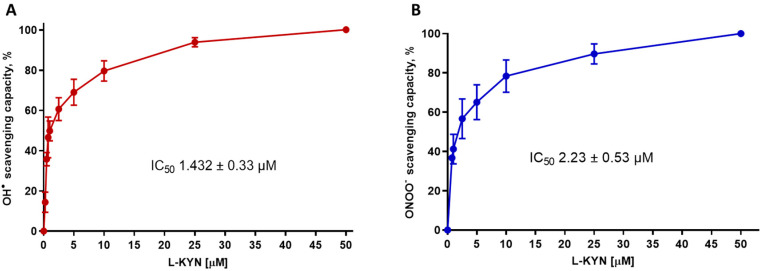
L-kynurenine (L-KYN) (1–50 µM) ability to scavenge (**A**) hydroxyl radical (^●^OH) and (**B**) peroxynitrite (ONOO^−^) in a chemical combinatory system. Data are presented as mean values ± S.E.M. of 6 experiments per concentration.

First, we determined the redox profile of L-KYN using non-tissue synthetic systems. Reactive oxygen species were specifically generated through combinatorial chemistry assays. L-KYN exhibited an efficient scavenging activity for ^●^OH and ONOO^−^, in a concentration-dependent manner, for example, the IC_50_ values calculated from the linear interpolation of the dose-response curve were 1.43 ± 0.33 µM and 2.23 ± 0.53 µM for ^●^OH and ONOO^−^, respectively, these values are close to the lowest tested concentration (Figure 2A,B, respectively). In contrast, L-KYN was unable to scavenge H_2_O_2_ and O_2_^•−^ in the same concentration range (0–50 µM).

**Figure 3:**
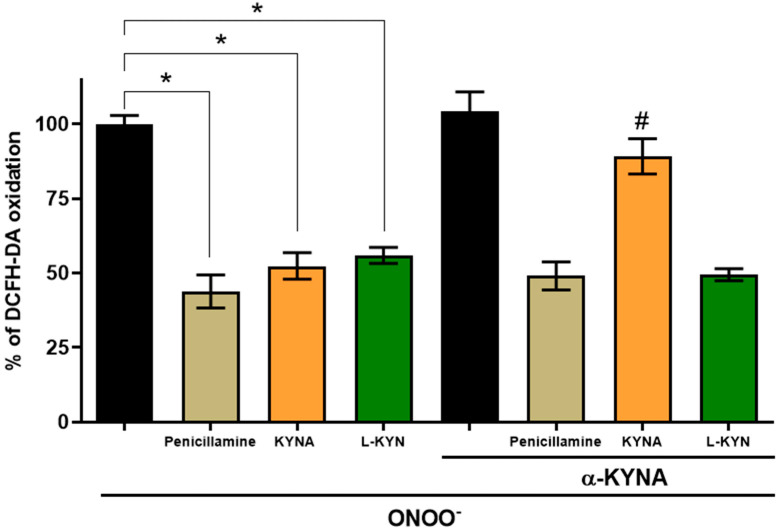
Effect of kynurenic acid (KYNA) antibody on the reduction of DCF-DA oxidation in the presence of ONOO^−^ scavengers: Penicillamine, KYNA and L-KYN. Anti-KYNA antibody was used as a tool to exclude the involvement of KYNA in the ONOO^−^ scavenging activity of L-KYN. The anti-KYNA antibody was added to the mixture reaction prior to penicillamine (300 µM), KYNA (600 µM) and L-KYN (2.2 µM). Data are presented as mean values ± S.E.M. of 8 independent experiments. * *p* < 0.05 vs. ONOO^−^ and ^#^ *p* < 0.05 vs. the corresponding scavenger without α-KYNA, based on the Kruskal–Wallis test with Dunn’s test for multiple pairwise comparisons.

Next, we designed an experiment to compare the ONOO^−^ scavenging activity between KYNA and L-KYN under the same conditions. We previously showed that L-KYN can produce KYNA non-enzymatically from its direct interaction with ONOO^−^ and characterized KYNA as a potential brain endogenous antioxidant and ROS scavenger [6]. Hence, to assess the L-KYN scavenging activity independently of KYNA formation we used a neutralization strategy for KYNA, similar to that previously reported for QUIN [42]; an anti-KYNA antibody was added to the chemical reaction to neutralize the scavenging effect of KYNA in the chemical combinatory assay for ONOO^−^. As a reference, the effect of L-KYN was compared with that of penicillamine, a specific ONOO^−^ scavenger. In the assay, the oxidation of DCF-DA induced by ONOO^−^ was markedly reduced by 56.2 ± 5.6%, 47.6 ± 5.5%, and 44 ± 2.7% by penicillamine (300 µM), KYNA (600 µM) and L-KYN (2.2 µM), respectively (Figure 3). However, the antioxidant effect of KYNA on DCF-DA oxidation was prevented when the anti-KYNA antibody was added to the reaction mixture (Table 1). In contrast, the scavenging effect of penicillamine and L-KYN remained the same; this would mean an independent effect of KYNA formation for L-KYN. The anti-KYNA antibody alone did not affect DCF-DA oxidation induced by ONOO^−^.

### 3.2. Effect of L-KYN on DNA and Protein Oxidative Degradation

**Figure 4:**
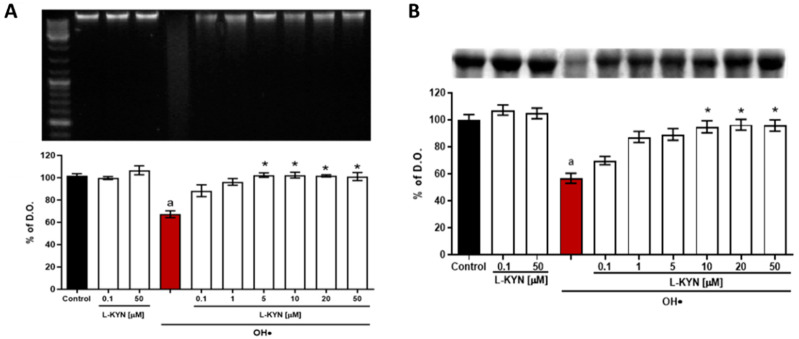
Protective effect of L-KYN on DNA and BSA oxidative degradation induced by ^•^OH. (**A**) Representative blot for stained-DNA detection (upper panel): line 1, molecular weight marker; line 2, DNA; line 3, DNA + L-KYN (0.1 µM); line 4, DNA + L-KYN (50 µM); line 5 DNA + ^●^OH; lines 6–11, DNA + ^●^OH + L-KYN (0.1, 1, 5, 10, 20, 50 µM, respectively) and quantitative representation for DNA detection (lower panel). (**B**) Representative Coomassie blue-stained-BSA gel (upper panel): line 1, BSA; line 2, BSA + L-KYN (0.1 µM); line 3, BSA + L-KYN (50 µM); line 4 BSA + ^●^OH; lines 5–10, BSA + ^●^OH + L-KYN (0.1, 1, 5, 10, 20, 50 µM, respectively) and quantitative representation for BSA detection (lower panel). Data are presented as mean ± S.E.M. of 3 independent experiments. ^a^ *p* < 0.05 ^•^OH vs. control; * *p* < 0.05 L-KYN with a specific concentration vs. ^•^OH, based on the Kruskal–Wallis test, with Dunn’s test for multiple pairwise comparisons.

The next step was to evaluate the effect of L-KYN on DNA and BSA degradation induced by ^•^OH (Figure 4A,B, respectively). L-KYN alone had no degradative effect on both DNA and BSA, even at the highest L-KYN concentration used (50 µM) compared to the respective control. A densitometric evaluation was employed for DNA and protein oxidative degradation (Figure 4A,B, respectively), whereby it became evident that L-KYN had a strong effect, preventing the oxidation of these molecules (Table 2). Figure 4A shows that the ^•^OH generator-system induced 32.7 ± 3% DNA degradation (Figure 4A, line 5). The DNA degradation was attenuated by L-KYN (0.1, 1, 5, 10, 20, 50 μM) in a concentration-dependent manner (L-KYN 5–50 µM). In the case of protein oxidation (Figure 4B), the ^•^OH generator-system induced 43.3 ± 3.7% of BSA degradation (line 4), which was prevented by L-KYN in a concentration-dependent manner (L-KYN 10–50 µM). These data confirm our previous observations from combinatorial chemistry assays showing that L-KYN can scavenge ^•^OH, here preventing DNA and protein oxidation.

### 3.3. Effect of L-KYN on ROS Production Induced by FeSO_4_ in Rat Brain Homogenates

**Figure 5:**
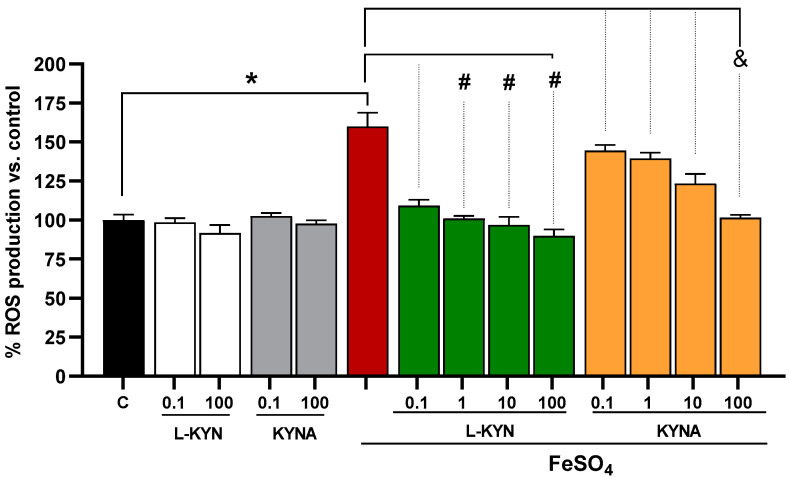
Effect of kynurenines on ROS production induced by FeSO_4_. Brain homogenates were incubated with L-KYN and KYNA (0–100 µM) with or without FeSO_4_ (10 µM). Data represent the mean ± S.E.M. of at least 5 independent experiments per concentration. * *p* < 0.05 FeSO_4_ vs. control; ^#^ *p* < 0.05 L-KYN with a specific concentration vs. FeSO_4_; and ^&^ *p* < 0.05 KYNA with a specific concentration vs. FeSO_4,_ based on the Kruskal–Wallis test with Dunn’s test for multiple pairwise comparisons.

We next tested the ability of L-KYN to reduce ROS production induced by FeSO_4_ (a known pro-oxidant that leads to ^•^OH production) in rat brain homogenates compared to those of KYNA at the same concentrations. As we can observe in Figure 5, the pro-oxidant FeSO_4_ substantially increased ROS production (60 ± 21.5% vs. control); however, this effect was diminished in brain homogenates by the co-incubation with L-KYN (0.1–100 µM; Table 3). Noticeably, even at the lowest L-KYN concentration, an antioxidant effect was achieved. When the co-incubation was carried-out with KYNA, ROS production was reduced gradually, and it became significant only at the highest concentration (100 µM). Therefore, in brain homogenates, L-KYN reduced ROS production more efficiently than KYNA (Table 3). L-KYN and KYNA alone did not induce ROS production.

### 3.4. Effect of L-KYN on Lipid Peroxidation and Cellular Dysfunction Induced by the Pro-Oxidant FeSO_4_ in Rat Brain Homogenates

**Figure 6:**
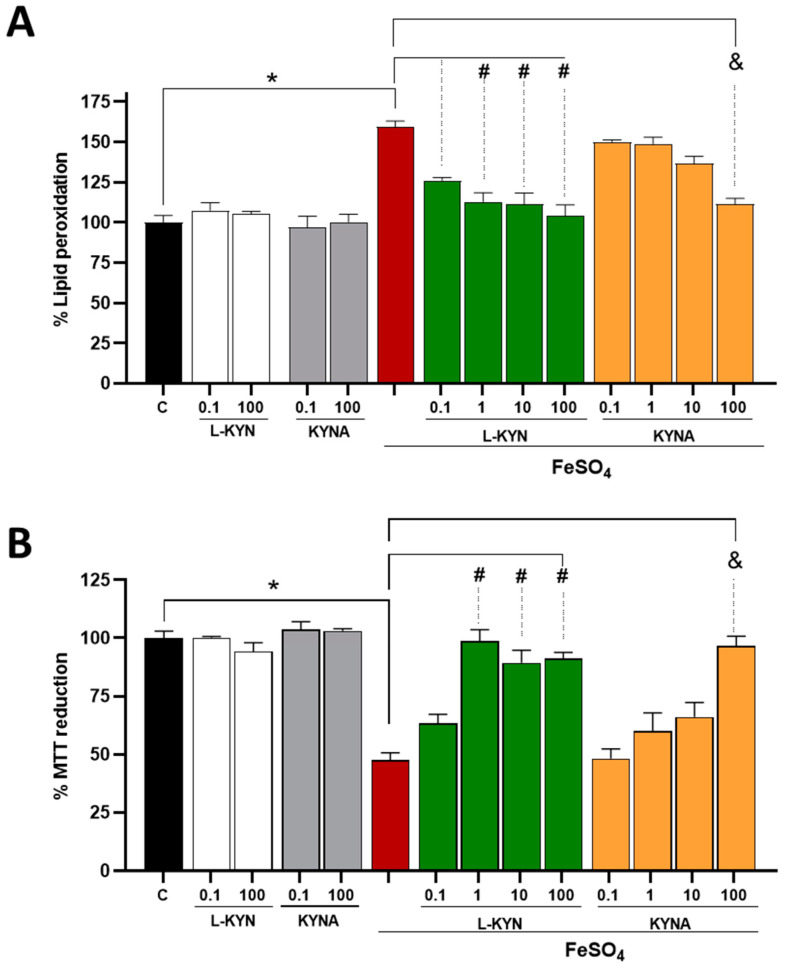
Effect of L-KYN on (**A**) LP and (**B**) cellular dysfunction induced by FeSO_4_. Brain homogenates were incubated with L-KYN and KYNA (0–100 µM) with or without FeSO_4_. Data represent the mean ± S.E.M. of at least 5 independent experiments per L-KYN or KYNA concentration. * *p* < 0.05 FeSO_4_ vs. control; ^#^ *p* < 0.05 L-KYN with a specific concentration vs. FeSO_4_; and ^&^ *p* < 0.05 KYNA with a specific concentration vs. FeSO_4_, based on the Kruskal–Wallis test, with Dunn’s test for multiple pairwise comparisons.

After showing that L-KYN efficiently reduced ROS production in brain homogenates exposed to FeSO_4_, we next analyzed other oxidative markers, namely LP and cellular dysfunction. The LP levels induced by the effect of FeSO_4_ in rat forebrain homogenates are shown in Figure 6A. FeSO_4_ significantly increased this oxidative marker (59.4 ± 9% vs. control), whereas the co-incubation with L-KYN markedly reduced the LP (56.2, 78.3, 81.1 and 93.1% for L-KYN 0.1, 1, 10 and 100 µM, respectively vs. FeSO_4_; Table 3). On the other hand, KYNA significantly decreased the LP induced by FeSO_4_ at the highest concentration tested (80.6 ± 8% vs. pro-oxidant). Figure 6B shows a decrease in MTT reduction, an indicator of cellular functionality in metabolically active cells, induced by the pro-oxidant agent, FeSO_4_ (52.6 ± 9% vs. control). L-KYN significantly attenuated this FeSO_4_-induced drop in cellular functionality at a concentration of 1 to 100 µM. In both tests, the incubation alone with L-KYN and KYNA was not different from the control.

### 3.5. Effect of Sub-Chronic Administration of L-KYN In Vivo on Redox Modulation

Once we characterized the scavenging effect of L-KYN using combinatorial chemistry assays, we evaluated its antioxidant properties in vitro, under oxidative conditions using rat brain homogenates. In view of several reports showing that prior or simultaneous administration of L-KYN attenuates cellular damage induced by neurotoxic agents in vivo, we hypothesized that the protective effect of L-KYN observed in these studies could be due to L-KYN redox properties, in addition to KYNA production; then we decided to evaluate whether a sub-chronic systemic administration of L-KYN (75 mg/kg for 5 days) could prevent the redox alterations induced ex vivo by different pro-oxidants in the brain tissue. After L-KYN sub-chronic administration, kynurenine pathway metabolites, L-KYN, 3-HK and KYNA, were measured. In parallel, the activity of the antioxidant enzymes (GR and GPx) and the content of GSH in brain tissue were also evaluated after sub-chronic administration of L-KYN.

An upward trend was observed in the levels of both KYNA and 3-HK in the brain homogenates from rats treated with L-KYN, whereas L-KYN brain levels were not different between Saline and L-KYN groups (Table 4).

**Figure 7:**
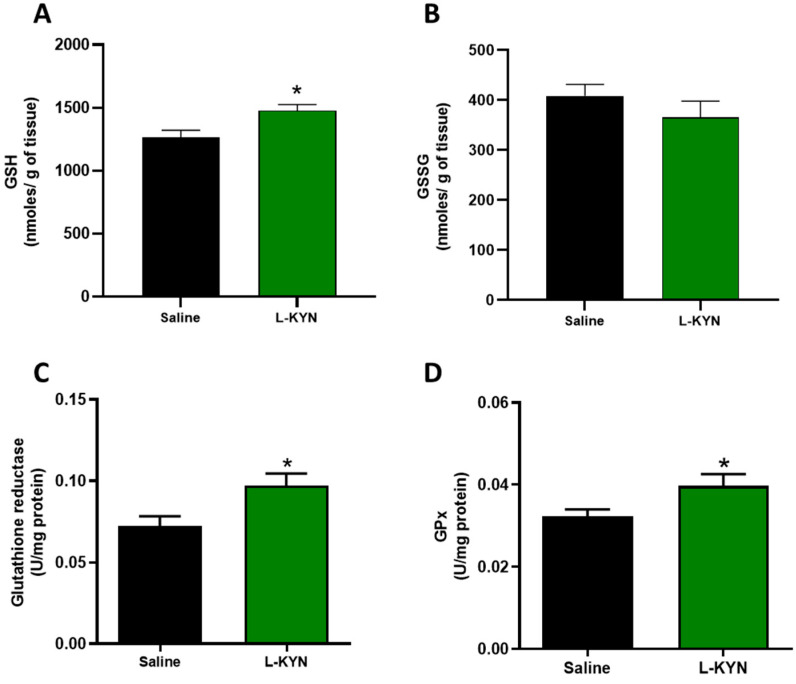
Brain levels of (**A**) GSH, (**B**) GSSG and (**C**) GR and D) GPx activity after sub-chronic L-KYN (75 mg/kg for 5 days) administration. Data are the mean ± S.E.M. (*n* = 5 per group). * *p* < 0.05 vs. Saline, based on Mann–Whitney test.

Furthermore, when the antioxidant markers were evaluated in the brain tissue, GSH levels increased slightly but significantly after the sub-chronic L-KYN administration, while brain GSSG levels showed a tendency to decrease (Figure 7). In agreement with these results, brain GR and GPx enzymatic activity increased in the group treated with L-KYN, thus suggesting that the sub-chronic administration of L-KYN in vivo (75 mg/kg for 5 days) significantly improves the antioxidant cellular environment in the brain tissue (Table 5).

**Figure 8:**
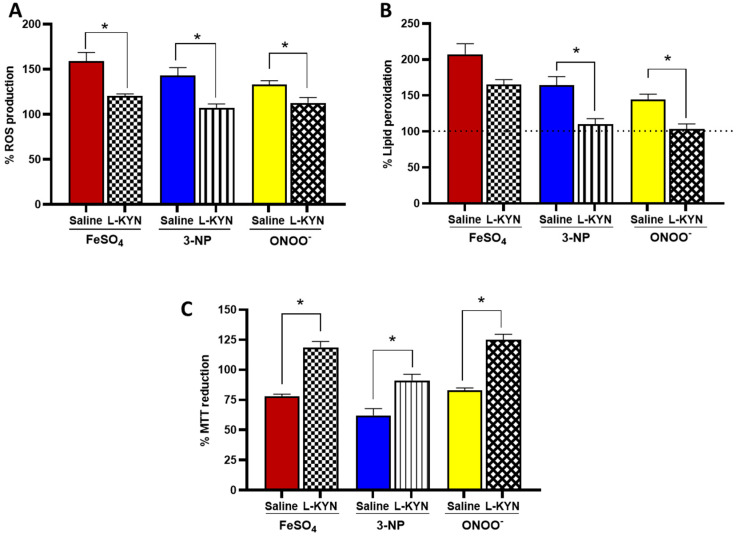
Effect of L-KYN treatment on (**A**) ROS, (**B**) LP and (**C**) MTT reduction in rat brain homogenates after the ex vivo exposure to pro-oxidants. After the last L-KYN administration, brain homogenates were incubated in the presence of FeSO_4_, 3-NP and ONOO^−^, then oxidative markers were determined considering the control as 100%. Data are the mean ± SEM (*n* = 5 per group); * *p* < 0.05 L-KYN vs. Saline based on the Mann–Whitney test for each pro-oxidant.

To further evaluate the enhanced antioxidant capacity in these brain tissues attributed to the systemic administration of L-KYN and whether this could mean a greater tolerance to pro-oxidant insults, we exposed the brain homogenates from Saline and L-KYN groups to FeSO_4_, 3-NP (succinate dehydrogenase inhibitor) and ONOO^−^. As shown in Figure 8A, ROS production increased 58.8 ± 10, 43.1 ± 9 and 32.9 ± 5% for FeSO_4_, 3-NP and ONOO^−^, respectively, in the Saline group. In contrast, ROS production in the brain homogenates from rats administrated with L-KYN was similar to control levels (120.5 ± 2, 106.9 ± 5 and 112.7 ± 6 for FeSO_4_, 3-NP and ONOO^−^, respectively). Hence, the exacerbated ROS production normally observed after exposure of the brain homogenate to each pro-oxidant tested was prevented by pre-treatment with L-KYN. Additionally, LP increased 107.4 ± 15, 64.3 ± 12 and 44.0 ± 8% when exposed to FeSO_4_, 3-NP and ONOO^−^ compared to the control in the Saline group (Figure 8B). Conversely, LP decreased significantly in the L-KYN group when considering 3-NP and ONOO^−^; a decreasing trend was observed for FeSO_4_. Finally, MTT reduction was also evaluated as an indicator of cellular function (Figure 8C). Formazan production decreased in the presence of all pro-oxidants tested in the Saline group (22.1 ± 2 for FeSO_4_, 37.8 ± 6 for 3-NP and 17.2 ± 2% for ONOO^−^ vs. control); however, this effect was diminished when these pro-oxidants were tested in brain homogenates from rats administrated with L-KYN (Table 6).

## 4. Discussion

L-KYN is an endogenous metabolite of tryptophan catabolism, and it is a crucial compound for the KP. Increasing evidence indicates an important role for L-KYN and its downstream metabolites in brain function. Downstream kynurenines such as KYNA have been studied more in depth, mainly because of its role in the control of the glutamatergic and cholinergic synaptic transmission. However, less is known about the specific properties of L-KYN itself. L-KYN levels have been measured in some neurodegenerative diseases, such as Alzheimer’s disease, in which peripheral levels have been shown to increase while those at the central nervous system level remained unchanged. L-KYN/TRP ratios have been also determined in Parkinson’s disease and increased L-KYN concentrations have been found in serum [43].

L-KYN has been extensively used as a pharmacological tool to increase KYNA levels in different experimental models. However, a time lag has been shown between kynurenine systemic administration and rising in KYNA levels in the brain. Recently, some studies in humans have shown a lack of increase in plasma KYNA after L-KYN administration, suggesting a relatively slow metabolism of L-KYN; this highlights the relevance of understanding the possible physiological effects of L-KYN at both the peripheral and central systems before producing the different downstream kynurenines [44].

The distinctive oxidative environment of the brain together with the important role of oxidative stress in neurodegenerative diseases and neuropsychiatric disorders has led us to focus on the role of oxidative mechanisms impacting the KP and to evaluate the redox profile of kynurenines. This study aimed to characterize the redox properties of L-KYN and its role in a physiological context where oxidative conditions are present. First, L-KYN scavenging ability was evaluated employing specific systems for ROS generation, in which L-KYN was shown to scavenge ^●^OH and ONOO^−^ (IC_50_: 1.432 ± 0.33 and 2.23 ± 0.53 µM, respectively), but not H_2_O_2_ and O_2_^●^¯. In addition, in combinatorial chemistry assays, L-KYN attenuated DNA and protein degradation induced by ^●^OH. However, considering that the scavenging effect of L-KYN in synthetic systems, could be attributed to the antioxidant properties of KYNA, [29,45]; we focused on excluding the KYNA participation in the scavenging effect of L-KYN. An anti-KYNA antibody was used to immediately block the KYNA produced by the direct interaction of L-KYN with peroxynitrite. Using this strategy, we showed that the scavenging effect of L-KYN remained unchanged in the presence of the anti-KYNA antibody while that of KYNA was abolished, indicating that the observed effect in combinatorial chemistry assays was mainly due to an L-KYN effect. This conclusion is supported by the fact that KYNA can scavenge ROS but with higher IC_50_ values (209 ± 7.4 and 598.4 ± 74.8 µM for ^●^OH and ONOO^−^, respectively) than L-KYN (1.4 ± 0.33 and 2.2 ± 0.5 µM for ^●^OH and ONOO^−^, respectively), indicating that L-KYN is a more efficient scavenger than KYNA [6]. These data agree with a study showing that kynurenine from P. *aeruginosa* inhibits ROS production of activated neutrophils, promoting bacterial survival. The same work demonstrated that L-KYN could scavenge O_2_^•¯^ and H_2_O_2_, but this kynurenine must be used at high concentrations (10–50 mM) to reach this effect [46]. Additionally, our combinatorial chemistry data are in accordance with a previous report demonstrating that L-KYN has a high capacity for auto-oxidation [25]. This means that is very likely that L-KYN can donate an electron and stabilize ROS, thus acting as an antioxidant [47]. Furthermore, it was previously shown that despite L-KYN not reacting efficiently with O_2_^•^^−^, it can react with ^●^OH radicals with a constant rate of 1.3 × 10^10^ M^−1^s^−1^ and 1.4 × 10^10^ M^−1^s^−1^ when is measured by pulse radiolysis and EPR-spin trapping, respectively [48,49].

Once the scavenging properties of L-KYN were described in combinatorial chemistry assays, its antioxidant ability in biological systems (rat brain homogenates) exposed to FeSO_4_ (a known pro-oxidant that leads to ^●^OH formation through the Fenton reaction) was explored. This pro-oxidant markedly increased ROS production and LP and decreased cell functionality; however, these alterations were prevented by adding L-KYN (1, 10 and 100 µM), starting from the lowest concentration used (0.1 µM) a protective trend can be appreciated. In comparison, when KYNA was used under the same in vitro conditions, the protective effect was reached with the highest concentration used (100 µM), suggesting that even if KYNA is produced by L-KYN (either by enzymatic transamination or by its interaction with ROS), the main antioxidant effect could be due to L-KYN or, once KYNA is formed, both antioxidant effects could be additive. These results are also in agreement with an earlier report showing that L-KYN decreased LP (~50%) induced by QUIN in brain homogenates. However, the protective effect was attributed to KYNA antagonism to NMDAr [50].

As mentioned before, L-KYN has been used as a neuroprotective strategy against excitotoxicity induced by QUIN [23], 6-OHDA [24], toxic soluble Aβ (25–35) [20], focal cerebral ischemia [18] and seizures induced by pentylenetetrazol [51]. The protective effect of using L-KYN as a pharmacological strategy has been largely attributed to KYNA production, a transamination product of L-KYN, based on its well-known NMDAr antagonist activity and antioxidant profile [6,52]. However, based on the results of this study, it can be suggested that the protective effect shown by L-KYN in these models can be partially attributed to its antioxidant properties, counteracting the redox alterations observed in these experimental models because of the oxidative stress, which can also trigger or exacerbate other deleterious events such as mitochondrial dysfunction, neuroinflammation, excitotoxicity, and cell death.

Finally, to determine if the sub-chronic administration of L-KYN favors the cellular environment with an enhanced antioxidant response against a pro-oxidant insult. We administered L-KYN systemically (75 mg/kg) for five days and further evaluated antioxidant markers in the brain tissue under basal conditions and when exposed to known pro-oxidants.

Our data showed that the antioxidant brain capacity increased, as evidenced by the increase in GSH content and the enhanced activity of GR and GPx enzymes. The fact that GSH (the most abundant thiol and main brain endogenous antioxidant), GR (the enzyme that reduces GSSG to GSH) and GPx (the enzyme that together with GSH neutralizes H_2_O_2_) activity increased suggests an enhanced antioxidant scenario in the brain tissue. This L-KYN effect could involve the induction of the nuclear factor-erythroid 2-related factor-2 (Nrf2), since within the downstream genes that are transcriptionally activated by this nuclear factor include those involved in GSH synthesis, such as *GCLC* as well as *GR* and *GPx* [53]. The enhanced antioxidant capacity of the brain by L-KYN administration was also tested ex vivo. Brain homogenates were exposed to three different pro-oxidants: (1) 3NP: a mitochondrial mycotoxin able to irreversibly inhibit succinate dehydrogenase, thereby causing prolonged energy impairment and ROS production [54]; (2) ONOO^−^: the product of the diffusion-controlled reaction of nitric oxide and superoxide radicals, with a significant oxidant and nucleophile capacity [55]; and (3) FeSO_4_: an ^●^OH generator and potent LP inducer. Under these pro-oxidant conditions, it was shown that those brain homogenates from rats administrated L-KYN attenuated the oxidative markers (ROS and LP) and the cellular dysfunction induced by all of the pro-oxidants. The antioxidant effect shown by L-KYN is due to its ability to scavenge ROS but also due to its ability to improve the cellular GSH-dependent antioxidant capacity.

Besides the antioxidant effects of L-KYN reported in this study, the role of the enantiomer D-kynurenine (D-KYN) as a source of L-KYN or even as an antioxidant metabolite cannot be ruled out, since scavenging properties for D-KYN have been demonstrated [56]. In this context, microbial activity of the D-amino oxidase and its contribution to the D-KYN-driven L-KYN pool has been reported [56] and the contribution of D-KYN administration in rising KYNA levels in mice and rats [45,56].

Although there is evidence that L-KYN possesses antioxidant and, in general, beneficial effects in diverse experimental models, it is also necessary to consider some studies that describe opposite results. In this line, a study showed that L-KYN can photo-oxidize cysteine, NADH and ascorbic acid, suggesting that these photooxidation processes may be responsible for the age-related depletion of reduced glutathione and/or formation of hydrogen peroxide in the lens [57]. Additionally, L-KYN acts on the aryl hydrocarbon receptor (AHR), affecting the metabolism of xenobiotics and promoting carcinogenesis; for example, it has been shown that L-KYN suppresses antitumor immune responses, promotes tumor cell survival and motility through the AHR in an autocrine/paracrine way [58]. It is clear that the KP, a highly conserved metabolic machinery, has evolved to perform relevant regulatory functions in the mammalian brain with a wide spectrum of actions. KP metabolites, such as L-KYN, can provide organ-specific protection depending on the cellular environment; however, it is necessary to elucidate the specific mechanisms by which these kynurenines perform their functional effects and how they change depending on the environment (inflammation, oxidative stress, energy deficit), as well as their impact on cellular metabolism. Considering the redox effects of L-KYN and knowing that L-KYN is the kynurenine most likely to modulate brain activity, these data provide evidence in understanding the specific mechanisms by which L-KYN regulates the antioxidant brain capacity. Although it has been suggested as a pharmacological strategy to increase KYNA levels and as a neuroprotective strategy in some pathologies (i.e., Parkinson’s Disease), little is known about L-KYN pharmacokinetics in humans. We still lack evidence of the basic properties of L-KYN. It was not until recently that the safety, tolerability, pharmacokinetics, and pharmacodynamics of L-KYN in humans were investigated [59]. This study showed that L-KYN was safe and well tolerated after intravenous infusion but did not change downstream kynurenine levels. Further characterization of L-KYN physiology in humans should be studied where different treatment strategies and larger sample sizes are implemented. Additionally, signaling pathways (activation of Nrf2) of antioxidant response and antioxidant enzymes such as superoxide dismutase and catalase, together with those enzymes involved in GSH synthesis, glutamate-cysteine ligase (GCL) and glutathione synthase (GSS), should be further determined to better understand the L-KYN antioxidant profile.

Further elucidation of the mechanisms that control and preserve the balance between peripheral and central L-KYN levels in mammals and the physiological effects that it produces should be considered in more depth.

## 5. Conclusions

This work shows that L-KYN has a dual antioxidant effect, it is a ROS scavenger, and it enhances the antioxidant capacity of the brain, thus providing protection mainly against oxidative damage through a mechanism that has not yet been elucidated. Future work should be focused on determining the degree of L-KYN involvement as a redox modulator in general and in those models in which it has already shown protection.

## Figures and Tables

**Table 1:** Effect of α-KYNA antibody on the reduction of DCF-DA oxidation in the presence of ONOO^−^ and its scavengers. Medians of the DCF-DA oxidation percentages and *p*-values associated with the effect of ONOO^−^ scavengers vs. ONOO^−^, using Dunn’s test for multiple pairwise comparisons.

	ONOO^−^				ONOO^−^ + α-KYNA			
		Penicillamine	KYNA	L-KYN		Penicillamine	KYNA	L-KYN
Median	98.17	47.22	52.38	59.78	103.4	46.68	91.76	50.08
*p*-value		0.0002	0.0237	0.0181	>0.999	0.0101	>0.999	0.0184

**Table 2:** The effect of L-KYN on ^●^OH-induced the DNA and Protein (BSA) oxidation. Medians of percentages of DNA and Protein oxidation and *p*-values associated with the effect of increasing concentrations of L-KYN vs. ^●^OH, using Dunn’s test for multiple pairwise comparisons.

			L-KYN + ^●^OH					
		^●^OH	0.1	1	5	10	25	50
**DNA**	Median	65.1	86.85	99.11	102.6	100.4	101.7	105.4
	*p*-value		>0.999	0.5319	0.0266	0.0248	0.0231	0.0266
**Protein**	Median	54.45	60.8	77.11	80.03	85.29	83.38	81.59
	*p*-value		>0.999	0.3936	0.2118	0.0301	0.0117	0.0179

**Table 3:** Effect of L-KYN and KYNA on ROS production, LP and MTT reduction induced by FeSO_4_. Medians of ROS production, PL and MTT reduction percentages and *p*-values associated with the effect of L-KYN and KYNA for each concentration vs. FeSO_4_, according to Dunn’s test for multiple pairwise comparisons.

		FeSO_4_	L-KYN (µM) + FeSO_4_				KYNA (µM) + FeSO_4_			
			0.1	1	10	100	0.1	1	10	100
**ROS** **production**	Median	153	108.4	100.8	101.5	85.83	141.6	139.9	120.4	99.65
	*p*-value		0.3352	0.0114	0.0065	0.0003	>0.9999	0.5333	0.0537	0.0032
**LP**	Median	156.2	126	109.7	109.9	107.6	151.3	150.3	137.1	113.8
	*p*-value		0.3684	0.0142	0.0065	0.0016	0.9272	0.7538	0.0518	0.0011
**MTT** **reduction**	Median	47.09	67.67	99.55	92.84	90.43	46.51	66.63	64.19	100.4
	*p*-value		>0.9999	0.0004	0.0057	0.0072	>0.9999	>0.9999	0.4408	0.0007

**Table 4:** Kynurenine metabolites brain levels.

	Saline	L-KYN	*p*-Value
**L-KYN** (pmoles/mg protein)	31.9 ± 8	32.1 ± 2	0.7000
**KYNA** (fmoles/mg protein)	60.4 ± 5	82.4 ± 7	0.1143
**3-HK** (pmoles/mg protein)	0.86 ± 0.2	1.39 ± 0.2	0.0649

**Table 5:** Effect of sub-chronic L-KYN administration on the brain antioxidant environment. Medians of GSH and GSSG levels, GR and GPx enzymatic activities and *p*-values associated with the effect of L-KYN compared to Saline, using the Mann–Whitney test.

		Saline	L-KYN
**GSH** **(nmoles/g of tissue)**	Median	1319	1429
	*p*-value		0.0041
**GSSG** **(nmoles/g of tissue)**	Median	414.7	375.4
	*p*-value		0.3829
**GR** **(U/mg protein)**	Median	0.07662	0.0965
	*p*-value		0.0320
**GPx** **(U/mg protein)**	Median	0.02981	0.0371
	*p*-value		0.0393

**Table 6:** Effect of sub-chronic administration of L-KYN on ROS production, reduction of LP and MTT induced by brain exposure to FeSO_4_, 3-NP and ONOO^−^ ex vivo. Medians of the percentages of ROS production, LP and MTT reduction and *p*-values associated with the effect of L-KYN for each pro-oxidant vs. Saline group, compared using the Mann–Whitney test.

		FeSO_4_		3-NP		ONOO^−^	
		Saline	L-KYN	Saline	L-KYN	Saline	L-KYN
**ROS** **production**	Median	152.0	118.9	134.4	106.8	132.8	113.8
	*p*-value		0.0004		0.0005		0.0317
**LP**	Median	200.3	161.1	158.8	108.6	142.4	99.62
	*p*-value		0.1143		0.0013		0.0238
**MTT** **reduction**	Median	76.60	119.1	62.57	91.02	84.17	127.1
	*p*-value		<0.0001		0.0317		0.0002

## Data Availability

The data is contained within the article.

## References

[1] Beadle G.W., Mitchell H.K., Nyc J.F. (1947). Kynurenine as an Intermediate in the Formation of Nicotinic Acid from Tryptophane by Neurospora. Proc. Natl. Acad. Sci. USA.

[2] Ciapala K., Mika J., Rojewska E. (2021). The Kynurenine Pathway as a Potential Target for Neuropathic Pain Therapy Design: From Basic Research to Clinical Perspectives. Int. J. Mol. Sci..

[3] Ishii T., Iwahashi H., Sugata R., Kido R. (1992). Oxidation of 3-hydroxykynurenine catalyzed by methemoglobin with hydrogen peroxide. Free Radic. Biol. Med..

[4] Iwahashi H., Kawamori H., Fukushima K. (1999). Quinolinic acid, alpha-picolinic acid, fusaric acid, and 2,6-pyridinedicarboxylic acid enhance the Fenton reaction in phosphate buffer. Chem. Biol. Interact..

[5] Platenik J., Stopka P., Vejrazka M., Stipek S. (2001). Quinolinic acid-iron(ii) complexes: Slow autoxidation, but enhanced hydroxyl radical production in the Fenton reaction. Free Radic. Res..

[6] Lugo-Huitron R., Blanco-Ayala T., Ugalde-Muniz P., Carrillo-Mora P., Pedraza-Chaverri J., Silva-Adaya D., Maldonado P.D., Torres I., Pinzon E., Ortiz-Islas E. (2011). On the antioxidant properties of kynurenic acid: Free radical scavenging activity and inhibition of oxidative stress. Neurotoxicol. Teratol..

[7] Pocivavsek A., Wu H.Q., Potter M.C., Elmer G.I., Pellicciari R., Schwarcz R. (2011). Fluctuations in endogenous kynurenic acid control hippocampal glutamate and memory. Neuropsychopharmacology.

[8] Pocivavsek A., Thomas M.A., Elmer G.I., Bruno J.P., Schwarcz R. (2014). Continuous kynurenine administration during the prenatal period, but not during adolescence, causes learning and memory deficits in adult rats. Psychopharmacology.

[9] Reyes-Ocampo J., Ramirez-Ortega D., Cervantes G.I., Pineda B., Balderas P.M., Gonzalez-Esquivel D., Sanchez-Chapul L., Lugo-Huitron R., Silva-Adaya D., Rios C. (2015). Mitochondrial dysfunction related to cell damage induced by 3-hydroxykynurenine and 3-hydroxyanthranilic acid: Non-dependent-effect of early reactive oxygen species production. Neurotoxicology.

[10] Schwarcz R., Stone T.W. (2017). The kynurenine pathway and the brain: Challenges, controversies and promises. Neuropharmacology.

[11] Goshima N., Wadano A., Miura K. (1986). 3-Hydroxykynurenine as O2-. scavenger in the blowfly, Aldrichina grahami. Biochem. Biophys. Res. Commun..

[12] Tanaka M., Toth F., Polyak H., Szabo A., Mandi Y., Vecsei L. (2021). Immune Influencers in Action: Metabolites and Enzymes of the Tryptophan-Kynurenine Metabolic Pathway. Biomedicines.

[13] Knox W.E., Mehler A.H. (1950). The conversion of tryptophan to kynurenine in liver. I. The coupled tryptophan peroxidase-oxidase system forming formylkynurenine. J. Biol. Chem..

[14] Ruddick J.P., Evans A.K., Nutt D.J., Lightman S.L., Rook G.A., Lowry C.A. (2006). Tryptophan metabolism in the central nervous system: Medical implications. Expert Rev. Mol. Med..

[15] Gal E.M., Sherman A.D. (1980). L-kynurenine: Its synthesis and possible regulatory function in brain. Neurochem. Res..

[16] Nozaki K., Beal M.F. (1992). Neuroprotective effects of L-kynurenine on hypoxia-ischemia and NMDA lesions in neonatal rats. J. Cereb. Blood Flow Metab..

[17] Robotka H., Sas K., Agoston M., Rozsa E., Szenasi G., Gigler G., Vecsei L., Toldi J. (2008). Neuroprotection achieved in the ischaemic rat cortex with L-kynurenine sulphate. Life Sci..

[18] Gigler G., Szenasi G., Simo A., Levay G., Harsing L.G., Sas K., Vecsei L., Toldi J. (2007). Neuroprotective effect of L-kynurenine sulfate administered before focal cerebral ischemia in mice and global cerebral ischemia in gerbils. Eur. J. Pharmacol..

[19] Sas K., Robotka H., Rozsa E., Agoston M., Szenasi G., Gigler G., Marosi M., Kis Z., Farkas T., Vecsei L. (2008). Kynurenine diminishes the ischemia-induced histological and electrophysiological deficits in the rat hippocampus. Neurobiol. Dis..

[20] Carrillo-Mora P., Mendez-Cuesta L.A., Perez-De La Cruz V., Fortoul-van Der Goes T.I., Santamaria A. (2010). Protective effect of systemic L-kynurenine and probenecid administration on behavioural and morphological alterations induced by toxic soluble amyloid beta (25–35) in rat hippocampus. Behav. Brain Res..

[21] Vecsei L., Beal M.F. (1990). Intracerebroventricular injection of kynurenic acid, but not kynurenine, induces ataxia and stereotyped behavior in rats. Brain Res. Bull..

[22] Miranda A.F., Sutton M.A., Beninger R.J., Jhamandas K., Boegman R.J. (1999). Quinolinic acid lesion of the nigrostriatal pathway: Effect on turning behaviour and protection by elevation of endogenous kynurenic acid in Rattus norvegicus. Neurosci. Lett..

[23] Santamaria A., Rios C., Solis-Hernandez F., Ordaz-Moreno J., Gonzalez-Reynoso L., Altagracia M., Kravzov J. (1996). Systemic DL-kynurenine and probenecid pretreatment attenuates quinolinic acid-induced neurotoxicity in rats. Neuropharmacology.

[24] Silva-Adaya D., Perez-De La Cruz V., Villeda-Hernandez J., Carrillo-Mora P., Gonzalez-Herrera I.G., Garcia E., Colin-Barenque L., Pedraza-Chaverri J., Santamaria A. (2011). Protective effect of L-kynurenine and probenecid on 6-hydroxydopamine-induced striatal toxicity in rats: Implications of modulating kynurenate as a protective strategy. Neurotoxicol. Teratol..

[25] Giles G.I., Collins C.A., Stone T.W., Jacob C. (2003). Electrochemical and in vitro evaluation of the redox-properties of kynurenine species. Biochem. Biophys. Res. Commun..

[26] Weiss G., Díez-Ruiz A., Murr C., Theur I., Fuchs D. (2002). Tryptophan Metabolites as Scavengers of Reactive Oxygen and Chlorine Species. Pteridines.

[27] Fontana M., Mosca L., Rosei M.A. (2001). Interaction of enkephalins with oxyradicals. Biochem. Pharmacol..

[28] Floriano-Sanchez E., Villanueva C., Medina-Campos O.N., Rocha D., Sanchez-Gonzalez D.J., Cardenas-Rodriguez N., Pedraza-Chaverri J. (2006). Nordihydroguaiaretic acid is a potent in vitro scavenger of peroxynitrite, singlet oxygen, hydroxyl radical, superoxide anion and hypochlorous acid and prevents in vivo ozone-induced tyrosine nitration in lungs. Free Radic. Res..

[29] Blanco Ayala T., Lugo Huitron R., Carmona Aparicio L., Ramirez Ortega D., Gonzalez Esquivel D., Pedraza Chaverri J., Perez de la Cruz G., Rios C., Schwarcz R., Perez de la Cruz V. (2015). Alternative kynurenic acid synthesis routes studied in the rat cerebellum. Front. Cell. Neurosci..

[30] Beckman J.S., Chen J., Ischiropoulos H., Crow J.P. (1994). Oxidative chemistry of peroxynitrite. Methods Enzymol..

[31] Crow J.P., Beckman J.S. (1996). The importance of superoxide in nitric oxide-dependent toxicity: Evidence for peroxynitrite-mediated injury. Adv. Exp. Med. Biol..

[32] Long L.H., Evans P.J., Halliwell B. (1999). Hydrogen peroxide in human urine: Implications for antioxidant defense and redox regulation. Biochem. Biophys. Res. Commun..

[33] Galano A., Macias-Ruvalcaba N.A., Medina Campos O.N., Pedraza-Chaverri J. (2010). Mechanism of the OH radical scavenging activity of nordihydroguaiaretic acid: A combined theoretical and experimental study. J. Phys. Chem. B.

[34] Kocha T., Yamaguchi M., Ohtaki H., Fukuda T., Aoyagi T. (1997). Hydrogen peroxide-mediated degradation of protein: Different oxidation modes of copper- and iron-dependent hydroxyl radicals on the degradation of albumin. Biochim. Biophys. Acta.

[35] Blanco Ayala T.B., Ramirez Ortega D.R., Ovalle Rodriguez P.O., Pineda B., Perez de la Cruz G.P., Gonzalez Esquivel D.G., Schwarcz R., Sathyasaikumar K.V., Jimenez Anguiano A.J., Perez de la Cruz V.P. (2021). Subchronic N-acetylcysteine Treatment Decreases Brain Kynurenic Acid Levels and Improves Cognitive Performance in Mice. Antioxidants.

[36] Mosmann T. (1983). Rapid colorimetric assay for cellular growth and survival: Application to proliferation and cytotoxicity assays. J. Immunol. Methods.

[37] Berridge M.V., Tan A.S. (1993). Characterization of the cellular reduction of 3-(4,5-dimethylthiazol-2-yl)-2,5-diphenyltetrazolium bromide (MTT): Subcellular localization, substrate dependence, and involvement of mitochondrial electron transport in MTT reduction. Arch. Biochem. Biophys..

[38] Carvalho-Silva M., Gomes L.M., de Pra S.D., Wessler L.B., Schuck P.F., Scaini G., de Bem A.F., Blum-Silva C.H., Reginatto F.H., de Oliveira J. (2020). Evidence of hippocampal astrogliosis and antioxidant imbalance after L-tyrosine chronic administration in rats. Metab. Brain Dis..

[39] Santana-Martinez R.A., Silva-Islas C.A., Fernandez-Orihuela Y.Y., Barrera-Oviedo D., Pedraza-Chaverri J., Hernandez-Pando R., Maldonado P.D. (2019). The Therapeutic Effect of Curcumin in Quinolinic Acid-Induced Neurotoxicity in Rats is Associated with BDNF, ERK1/2, Nrf2, and Antioxidant Enzymes. Antioxidants.

[40] Andersen H.R., Nielsen J.B., Nielsen F., Grandjean P. (1997). Antioxidative enzyme activities in human erythrocytes. Clin. Chem..

[41] Lowry O.H., Rosebrough N.J., Farr A.L., Randall R.J. (1951). Protein measurement with the Folin phenol reagent. J. Biol. Chem..

[42] Sundaram G., Brew B.J., Jones S.P., Adams S., Lim C.K., Guillemin G.J. (2014). Quinolinic acid toxicity on oligodendroglial cells: Relevance for multiple sclerosis and therapeutic strategies. J. Neuroinflamm..

[43] Torok N., Tanaka M., Vecsei L. (2020). Searching for Peripheral Biomarkers in Neurodegenerative Diseases: The Tryptophan-Kynurenine Metabolic Pathway. Int. J. Mol. Sci..

[44] Detrait M., Pesse M., Calissi C., Bouyon S., Brocard J., Vial G., Pepin J.L., Belaidi E., Arnaud C. (2021). Short-term intermittent hypoxia induces simultaneous systemic insulin resistance and higher cardiac contractility in lean mice. Physiol. Rep..

[45] Ramos-Chavez L.A., Lugo Huitron R., Gonzalez Esquivel D., Pineda B., Rios C., Silva-Adaya D., Sanchez-Chapul L., Roldan-Roldan G., Perez de la Cruz V. (2018). Relevance of Alternative Routes of Kynurenic Acid Production in the Brain. Oxid. Med. Cell. Longev..

[46] Genestet C., Le Gouellec A., Chaker H., Polack B., Guery B., Toussaint B., Stasia M.J. (2014). Scavenging of reactive oxygen species by tryptophan metabolites helps Pseudomonas aeruginosa escape neutrophil killing. Free Radic. Biol. Med..

[47] Zhuravlev A.V., Zakharov G.A., Shchegolev B.F., Savvateeva-Popova E.V. (2016). Antioxidant Properties of Kynurenines: Density Functional Theory Calculations. PLoS Comput. Biol..

[48] Matuszak Z., Reszka K., Chignell C.F. (1997). Reaction of melatonin and related indoles with hydroxyl radicals: EPR and spin trapping investigations. Free Radic. Biol. Med..

[49] Atherton S.J., Dillon J., Gaillard E.R. (1993). A pulse radiolysis study of the reactions of 3-hydroxykynurenine and kynurenine with oxidizing and reducing radicals. Biochim. Biophys. Acta.

[50] Rios C., Santamaria A. (1991). Quinolinic acid is a potent lipid peroxidant in rat brain homogenates. Neurochem. Res..

[51] Nemeth H., Robotka H., Kis Z., Rozsa E., Janaky T., Somlai C., Marosi M., Farkas T., Toldi J., Vecsei L. (2004). Kynurenine administered together with probenecid markedly inhibits pentylenetetrazol-induced seizures. An electrophysiological and behavioural study. Neuropharmacology.

[52] Pocivavsek A., Notarangelo F.M., Wu H.-Q., Bruno J.P., Schwarcz R., Pletnikov M.V., Waddington J.L. (2016). Chapter 25—Astrocytes as Pharmacological Targets in the Treatment of Schizophrenia: Focus on Kynurenic Acid. Handbook of Behavioral Neuroscience.

[53] Zalachoras I., Hollis F., Ramos-Fernandez E., Trovo L., Sonnay S., Geiser E., Preitner N., Steiner P., Sandi C., Morato L. (2020). Therapeutic potential of glutathione-enhancers in stress-related psychopathologies. Neurosci. Biobehav. Rev..

[54] Sandhir R., Mehrotra A. (2013). Quercetin supplementation is effective in improving mitochondrial dysfunctions induced by 3-nitropropionic acid: Implications in Huntington’s disease. Biochim. Biophys. Acta.

[55] Radi R. (2013). Peroxynitrite, a stealthy biological oxidant. J. Biol. Chem..

[56] Tanaka M., Bohar Z., Vecsei L. (2020). Are Kynurenines Accomplices or Principal Villains in Dementia? Maintenance of Kynurenine Metabolism. Molecules.

[57] Reszka K.J., Bilski P., Chignell C.F., Dillon J. (1996). Free radical reactions photosensitized by the human lens component, kynurenine: An EPR and spin trapping investigation. Free Radic. Biol. Med..

[58] Opitz C.A., Litzenburger U.M., Sahm F., Ott M., Tritschler I., Trump S., Schumacher T., Jestaedt L., Schrenk D., Weller M. (2011). An endogenous tumour-promoting ligand of the human aryl hydrocarbon receptor. Nature.

[59] Al-Karagholi M.A., Hansen J.M., Abou-Kassem D., Hansted A.K., Ubhayasekera K., Bergquist J., Vecsei L., Jansen-Olesen I., Ashina M. (2021). Phase 1 study to access safety, tolerability, pharmacokinetics, and pharmacodynamics of kynurenine in healthy volunteers. Pharmacol. Res. Perspect..

